# Graph-based analysis of EEG for schizotypy classification applying flicker Ganzfeld stimulation

**DOI:** 10.1038/s41537-023-00395-4

**Published:** 2023-09-21

**Authors:** Ahmad Zandbagleh, Sattar Mirzakuchaki, Mohammad Reza Daliri, Alexander Sumich, John D. Anderson, Saeid Sanei

**Affiliations:** 1 School of Electrical Engineering, Iran University of Science and Technology, Tehran, Iran; 2 Division of Psychology, Nottingham Trent University, Main Campus, Nottingham, UK; 3 School of Science and Technology, Nottingham Trent University, Clifton Lane, Nottingham, UK

**Keywords:** Human behaviour, Neural circuits

## Abstract

Ganzfeld conditions induce alterations in brain function and pseudo-hallucinatory experiences, particularly in people with high positive schizotypy. The current study uses graph-based parameters to investigate and classify brain networks under Ganzfeld conditions as a function of positive schizotypy. Participants from the general population (14 high schizotypy (HS), 29 low schizotypy (LS)) had an electroencephalography assessment during Ganzfeld conditions, with varying visual activation (8 frequencies of random light flicker) and soundscape-induced mood (neutral, serenity, and anxiety). Weighted functional networks were computed in six frequency sub-bands (delta, theta, alpha-low, alpha-high, beta, and gamma) as a function of light-flicker frequency and mood. The brain network was analyzed using graph theory parameters, including clustering coefficient (CC), strength, and global efficiency (GE). It was found that the LS groups had higher CC and strength than the HS groups, especially in bilateral temporal and frontotemporal brain regions. Moreover, some decreases in CC and strength measures were found in LS groups among occipital and parieto-occipital brain regions. LS groups also had significantly higher GE in all Ganzfeld conditions compared to the HS groups. The random under-sampling boosting (RUSBoost) algorithm achieved the best classification performance with an accuracy of 95.34%, specificity of 96.55%, and sensitivity of 92.85% during an anxiety-induction Ganzfeld condition. This is the first exploration of the relationship between brain functional state changes under Ganzfeld conditions in individuals who vary in positive schizotypy. The accuracy of graph-based parameters in classifying brain states as a function of schizotypy is shown, particularly for brain activity during anxiety induction, and should be investigated in psychosis.

## Introduction

Positive schizotypy (or psychosis proneness) refers to personality, cognitive, and perceptual traits akin to psychosis but observed at subdiagnostic threshold in the general population^1–3^. People high in positive schizotypy are more likely to experience pseudo-hallucinations under Ganzfeld conditions^4^, that is, a uniform and unstructured perceptual field created by translucent eye covers and often combined with random stimulation (e.g., light flicker at various frequencies)^5,6^. Such conditions induce pseudo-hallucinatory imagery and may alter states of consciousness in the general population^6,7^, mostly in people high in positive schizotypy. Recent work investigating brain function, using electroencephalography (EEG), under Ganzfeld conditions and in relation to psychosis proneness has implicated alterations in the production of occipital alpha activity^4^. On the other hand, the occurrence of psychotic experiences in the general population, with concepts such as schizotypy, is more frequent than schizophrenia diagnosis^8^. Treatment delay in the psychosis-like symptoms has led to poorer outcomes in psychosis^9^. So, drawing the bridge between research into the neurobiology of schizophrenia and psychosis proneness leads psychologists to develop novel psychosocial interventions^10^. Our group is developing signal processing techniques that facilitate the estimation of brain complexity measures, such as connectivity^11^. The current study applies these methods to understand and classify neurophysiological function in relation to positive schizotypy during Ganzfeld conditions.

Schizotypy has been studied using resting state^12–16^ and time-locked EEG^17–20^. For example, alteration in frontal alpha asymmetry^12^, reduced right frontal P300^17^, lower occipital alpha power under Ganzfeld conditions^4^, lower gamma power at rest^15^, significantly lower ERP amplitude in some brain regions^19^, lower occipital alpha and frontal theta power during praise and criticism^20^ have been associated with high positive schizotypy. Connectivity in complex brain networks^21^ has been examined in relation to psychopathology and schizotypy using graph theory^22,23^. Graph theoretical analysis is a powerful method to evaluate and characterize the topological structures and functional brain networks. According to graph theory, complex brain networks are modeled mathematically as a graph which includes sets of nodes and edges that represent EEG channels and their correlations^24^. Converging evidence confirms that the connectivity of brain networks is specifically influenced by psychiatric disorders (e.g., schizophrenia patients showed reduced connectivity strength, characteristic path length, and clustering coefficient (CC) compared to healthy control individuals and the gray matter lesions are concentrated both in frontal and temporal cortical hubs in schizophrenia patients^25^). Although schizotypy symptoms are less clear, compared to negative schizotypy and healthy controls, positive schizotypy is associated with a smaller difference in CC and node strength in frontal-occipital and central-occipital regions (indicating more widespread networks), whilst negative schizotypy was associated with a larger difference in central-occipital regions (indicating localized occipital focuses in brain network)^22^.

Hardly any analytically detailed machine learning approach for schizotypy can be seen in the literature. One study^26^ attempting to classify brain states in schizotypy and healthy control groups during an audio-visual emotion task using a shrinkage linear discriminant algorithm, achieved a zero false-positive rate. Trajkovic et al.^16^. classified high and low schizotypy (LS) group with 74.3% accuracy by combining measures of right posterior alpha activity (speed and amplitude) and connectivity (right frontoparietal alpha). Higher accuracy (89.21%) was obtained by examining effective brain connectivity, namely directed transfer function, using multivariate autoregressive coefficients^27^. Even higher accuracy (93.1%) was obtained using machine learning methods to differentiate schizotypy-associated brain states based on the P300 subcomponents derived using tensor factorization during an auditory oddball task^18^.

The current study applied a graph-based representation of phase locking value (PLV) to EEG data recorded under Ganzfeld conditions^4^ in order to assess functional brain connectivity and determine EEG-based brain networks in high schizotypy (HS) and LS groups. Then, two steps are conducted for statistical analysis. In the first step, weighted functional brain networks are compared statistically between the two groups before thresholding. In the second step, a statistical comparison of some graph parameters, including CC, strength, and global efficiency (GE), is performed using weighted connectivity matrices after thresholding. Finally, our proposed method is evaluated using influential graph-based features and some conventional classifiers, including decision tree (DT), support vector machine (SVM), K-nearest neighbor (KNN), linear discriminant analysis (LDA), and random under-sampling boosting (RUSBoost). To the best of our knowledge, this is the first study that examines the relationship between graph theory parameters of weighted networks associated with Ganzfeld stimulation and positive schizotypy. It is hypothesized that diffuse patterns of brain connectivity, as well as a lack of synchronization between different brain regions, can be utilized to classify HS and LS groups based on previous research on more advanced psychosis such as schizophrenia or Alzheimer’s^25,28,29^. For exploratory purposes, we also measured the differences between HS and LS in certain graph-based parameters in three Ganzfeld conditions.

## Materials and methods

### Participants

The current study uses the dataset reported by Sumich et al.^4^, which received ethical approval from the College of Business, Law and Social Science Research Ethics Committee at Nottingham Trent University (No. 2013/05). Data were collected through the BIAL Fellowship Programme [66/12] to Alexander Sumich, kindly supported by Fundação BIAL, Portugal. Forty-six participants (16 males, aged 18–57 years, mean = 24.15 ± 9.26) were recruited from the general and academic populations. All participants completed self-report scales for trait mental imagery (shortened Betts’ questionnaire upon mental imagery^30^) and perceptual anomalies to assess positive schizotypy (Cardiff anomalies perceptual scale, CAPS^31^) before taking part in the Ganzfeld imagery task. In addition, they provided informed consent prior to their involvement in the study. Based on the CAPS scores, participants were divided into LS (*n* = 29) and HS (*n* = 14) groups. Table 1 indicates the demographic characteristics and scores based on the CAPS.

**Table 1:** Demographic characteristics and scores on the CAPS.

	HS (*n* = 14)	LS (*n* = 29)	F/Chi-square (*df*)	*p*-value
Age	20.42 (2.53)	25.37 (10.04)	3.26 (1,42)	0.078
Sex (male/female)	7/7	8/21	2.088 (1)	0.148
CAPS	14.92 (3.58)	2.93 (2.25)	180.45 (1,42)	≪0.001

### Ganzfeld imagery task

In order to induce Ganzfeld conditions, participants wore plastic whiteout goggles to create a homogeneous visual field. An apparatus comprising 300 strips of red, green, and blue light-emitting diodes (RGB-LEDs) mounted on a curved panel (width: 51 cm, height: 32 cm) was positioned in front of the participants. An LM324N (quad Op-Amp) was used to control the panel of LEDs by converting auditory waves into light signals. Red light flickers were presented at eight target frequencies, including the lower band (8–16 Hz with 2 Hz step length) and higher band (20, 22, and 24 Hz). In addition, three emotion-induction blocks were created using soundscapes (serenity, anxiety) and pink noise (neutral) presented through headphones. In each block, the same sequence of eight target frequencies was used. Each frequency lasted for 30 s and was repeated 5 times per block. In each block, participants indicated, with a button press, whenever they observed simple (e.g., spirals, lines, and zigzags) or complex images (e.g., objects, animals, and human body parts)^32^. Further information about the designed instruments and the Ganzfeld imagery task has been explained in^4^.

### EEG recording and preprocessing

**Fig. 1:**
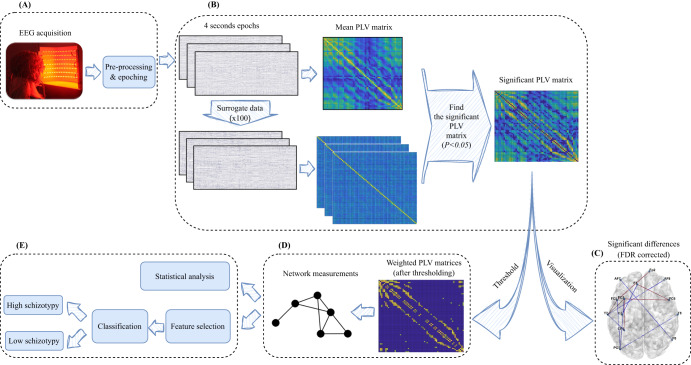
The block diagram of the overall proposed method for the statistical analysis and classification of LS and HS individuals. A Data acquisition and pre-processing block. B Functional brain network estimation block. C Visualization of significant differences before thresholding. D Mapping connectivity estimates to a graph after thresholding. E Statistical analysis and classification block.

EEG was recorded throughout using BioSemi Active-Two amplifier (64 channels, sampling rate = 2048 Hz; Biosemi Inc., Amsterdam, Netherlands). The artifacts were reduced using the EEGLAB toolbox^33^. After down-sampling to 256 Hz, a common average reference was performed to re-reference the data channels. All the channels were bandpass filtered using a 0.5–40 Hz finite impulse responses (FIR) zero-phase filter. Additionally, artifactual time points were removed by visual inspection. Then, an independent component analysis (ICA) using the logistic Infomax ICA algorithm^34^ was implemented to remove EOG artifacts. Finally, 20 non-overlapping 4-s epochs were extracted from the continuous EEG data from the onset of each Ganzfeld target frequency. Consequently, both statistical and machine-learning approaches were conducted by exploiting functional brain networks. The analysis framework of our proposed method is shown in Fig. 1. As shown in Fig. 1, each epoch of 4 s was used to estimate PLV, and their average was statistically compared with the surrogated PLV. A surrogate algorithm was then applied to eliminate weak or spurious connections. The actual PLV values were subsequently used for further analysis, including graph theory and machine learning.

### Brain network analysis

The functional brain connectivity based on the PLV is estimated, as a phase synchronization measure, between pairs of EEG channels in six frequency bands, including delta (2–4 Hz), theta (4–8 Hz), alpha-low (8–10 Hz), alpha-high (10–13), beta (13–30 Hz), and gamma (30–40 Hz). Furthermore, some indices are estimated based on graph theory for brain network quantification. Subsequently, the two schizotypy groups are evaluated based on these measures. The differences between HS and LS groups are estimated using Welch’s *t*-test (two-sample)^35,36^. A one-sample Kolmogorov–Smirnov^37^ is applied for the normality test. Then, the false discovery rate (FDR) correction of 0.05 is used to control the effect of multiple comparisons. A *p-*value lower than 0.05 is considered a significant level for all tests. The PLV details and graph parameters are presented in the following subsections.

### Phase synchrony analysis

Suitable techniques have been developed to assess the phase synchronization between channel (or brain region) pairs^11,24^. PLV, as a widely used method, is used for assessing the statistical dependency of neural time series in the frequency domain^38^.

Before computing the PLV, an FIR bandpass filter is employed to acquire narrowband signals for each conventional EEG frequency band. Then, the Hilbert transform is used for estimating the instantaneous phase time series. The complex analytic form of signals ${x}_{1}\left(t\right)$ and ${x}_{2}\left(t\right)$ can be obtained as:^38^

1 $${z}_{1}\left(t\right)={x}_{1}\left(t\right)+{jHT}({x}_{1}\left(t\right))$$

2 $${z}_{2}\left(t\right)={x}_{2}\left(t\right)+{jHT}({x}_{2}\left(t\right))$$

where ${z}_{1}\left(t\right)$ and ${z}_{2}\left(t\right)$ are the complex analytic form of signals ${x}_{1}\left(t\right)$ and ${x}_{2}\left(t\right)$, respectively. ${HT}$ represents the Hilbert transform operator and defined as^11,38^:

3 $${HT}\left({x}_{i}\left(t\right)\right)=\frac{1}{\pi }\,{\int }_{-{{\infty }}}^{+{{\infty }}}\frac{{x}_{i}\left(\tau \right)}{t-\tau }{\rm{d}}\tau$$

The relative phase between two analytic signals ${z}_{1}\left(t\right)$ and ${z}_{2}\left(t\right)$ is defined as^38^:

4 $${\varphi }_{12}\left(t\right)=\arctan \left(\frac{{HT}({x}_{1}\left(t\right))}{{x}_{1}}\right)-\arctan \left(\frac{{HT}({x}_{2}\left(t\right))}{{x}_{2}}\right)$$

Considering the above definitions, the PLV can be computed as^11,38^:

5 $${\rm{PLV}}\left({x}_{1},{x}_{2}\right)=\left|\frac{1}{N}\mathop{\sum }\limits_{k=0}^{N-1}{e}^{i{\varphi }_{12}\left({t}_{k}\right)}\right|=\sqrt{{\left[\frac{1}{N}\mathop{\sum }\limits_{k=0}^{N-1}\sin ({\varphi }_{12}\left({t}_{k}\right))\right]}^{2}+{\left[\frac{1}{N}\mathop{\sum }\limits_{k=0}^{N-1}\cos ({\varphi }_{12}\left({t}_{k}\right))\right]}^{2}}$$

where *N* is the number of time points. The PLV value varies between zero and one. Zero and one values represent unsynchronized phases and perfect synchronization, respectively.

A surrogate data technique^39^ is used to identify statistically significant connectivity values and remove fake connections. The surrogate values are calculated in each individual and condition by shuffling the phase information of the signal ${x}_{1}\left(t\right)$ while leaving the phase of signal ${x}_{2}\left(t\right)$ intact. So, the surrogate PLV can be defined as^40^:

6 $${{\rm{PLV}}}_{{surr}}\left(t\right)=\frac{1}{M}\mathop{\sum }\limits_{m=1}^{M}\left|\frac{1}{N}\mathop{\sum }\limits_{k=0}^{N-1}{e}^{i\left(\right.{\varphi }_{{x}_{1}}\left({t}_{k}\right)-{\varphi }_{{x}_{2},{perm}}\left({t}_{k,m}\right)}\right|$$

where ${\varphi }_{{x}_{2},{perm}}$ is the permuted phase of signal ${x}_{2}\left(t\right)$. Also, *M* indicates the number of surrogates. In this study, 100 surrogates were used to identify statistically significant PLV values. The connections were evaluated by comparing original PLV values to obtained surrogate PLV values. So, only the connections above the significance threshold of 0.05 were selected, while any non-significant PLV values were set to zero.

### Graph parameters

In this study, graph theory parameters were determined after constructing the undirected weighted brain network. In graph theory, the brain is represented mathematically as a connected graph composed of nodes and links in which the nodes represent EEG channels (or brain regions), and the links between the nodes represent the connectivity values^24,41^. The threshold value can be chosen based on data distributions or the sparsity of the adjacency matrix^42^. Selecting an optimal threshold value to eliminate insignificant links and to keep the same density in the graphs is still an open question^43^. To avoid the arbitrary selection of thresholds, it is advisable to apply a range of thresholds in practice^44^. To achieve this goal, sparsity thresholds ranging from 0.01 to 0.5 with 0.01 step length were considered in the current study. This range is chosen based on statistical results as well as the minimum density observed across all networks for all individuals in all Ganzfeld frequency bands. It is worth noting that we employed a surrogate technique to remove fake and weak connectivities beforehand to ensure the validity of our findings. It is important to note that graphs can be investigated at different levels of scale, with specific measures capturing attributes at either local or global scales. Nodal measures refer to simple statistics that capture attributes of individual nodes, while global measures express network-wide attributes that reflect the overall structure and connectivity of the entire graph^45^. Then, two local graph parameters were calculated from the weighted adjacency matrices, namely strength as a basic measure and CC as a measure of segregation. In addition, a global graph parameter was determined from the same matrices, namely GE, as a measure of integration^21^. To estimate these parameters, the Brain Connectivity Toolbox was used^21^. Also, the visualization of brain space graphs was performed using the BrainNet Viewer toolbox^46^. Concerning the statistical test results, a fixed density level of 0.08 is used for extracting the graph parameters for subsequent analysis.

The degree of each node is determined by the number of links connected to it, which is equal to the number of its immediate neighbors. The strength of a node is defined as the sum of link weights connected to the node, which is equal to the weighted degree of the node. So, the node strength is expressed as^21^:

7 $${s}_{i}^{w}=\sum _{j\in N}{W}_{{ij}}$$

where *i* and *N* show the node’s index and the number of nodes, respectively. ${W}_{{ij}}$ is the weighted connection link connecting node *i* to its neighbor *j*.

Measures of segregation that describe the presence of clusters within a network are interpreted as an organization of segregated neural processing in functional networks. CC, as a measure of segregation, is determined as the portion of a particular node’s neighbors that are also neighbors of each other. In other words, the fraction of triangles around each node denotes CC. The weighted CC of node *i* is defined as^21^:

8 $${{CC}}_{i}^{w}=\frac{1}{N}\mathop{\sum }\limits_{i=1}^{N}\frac{2{t}_{i}^{w}}{{k}_{i}({k}_{i}-1)}$$

where ${k}_{i}$ is the degree of node *i*. The geometric mean of triangles around node *i* (i.e., ${t}_{i}^{w}$) is written as^21^:

9 $${t}_{i}^{w}=\frac{1}{2}\sum _{j,h}{({W}_{{ij}}{W}_{{jh}}{W}_{{ih}})}^{\frac{1}{3}}$$

Considering the concept of a path, measures of integration in the brain can be used for evaluating the ability to rapidly integrate the information among the brain regions. The GE is determined as the average of the shortest path length inverse in networks. Thus, the network-weighted GE is calculated as^21^:

10 $${{GE}}^{w}=\frac{1}{N(N-1)}\mathop{\sum }\limits_{i=1}^{N}\mathop{\sum }\limits_{j=1,i\ne j}^{N}\frac{1}{{d}_{p}^{w}(i,j)}$$

where ${d}_{p}^{w}$ refers to the shortest weighted path between the node *i* and the node *j*.

### Feature extraction and classification

Three graph theory parameters, including CC, strength, and GE, were derived from the PLV adjacency matrix in the six mentioned frequency bands. These features were acquired for each of the three emotion inductions (i.e., neutral, serenity, and anxiety), while each emotion induction was divided into eight Ganzfeld target frequencies. Then, the informative and significant features were chosen using Welch’s *t*-test as a filtering technique for feature selection. So, the selected features are the most discriminative ones to use in the classification models^47^. Finally, the informative features to distinguish between schizotypy groups were evaluated using leave-one-out cross-validation (LOO-CV) via some conventional classifiers, including DT, SVM, KNN, LDA and RUSBoost^48^. In the LOO-CV performance evaluation approach, one individual is used for the test process, while other individuals are used for the training procedure^49^.

RUSBoost, as a fast hybrid boosting classification algorithm, is highly recommended in the literature for the performance evaluation of imbalanced data^50^. RUSBoost integrates RUS and AdaBoostM1 algorithm. In this algorithm, resampling is executed based on the weights given to samples contained in the training datasets. So, RUS randomly excludes samples from the majority class until the desired balance between the class distributions is achieved. Additionally, This outperforms AdaBoostM1, which is a boosting algorithm that uses an ensemble method with a DT as a learner^50^. So, it sequentially trains a next learner model on the samples misclassified by previous learners.

The assessment of the model performance was evaluated by accuracy, sensitivity, specificity, and F1-score measures^18^. The number of informative features was chosen based on a trial-and-error approach to minimize the classification error.

## Results

### Statistical analysis

The statistical analysis was performed in two steps. Firstly, we compared the functional connectivity networks between the two schizotypy groups before applying the threshold. Then, we compared the graph parameters (after thresholding) between the two schizotypy groups.

**Fig. 2:**
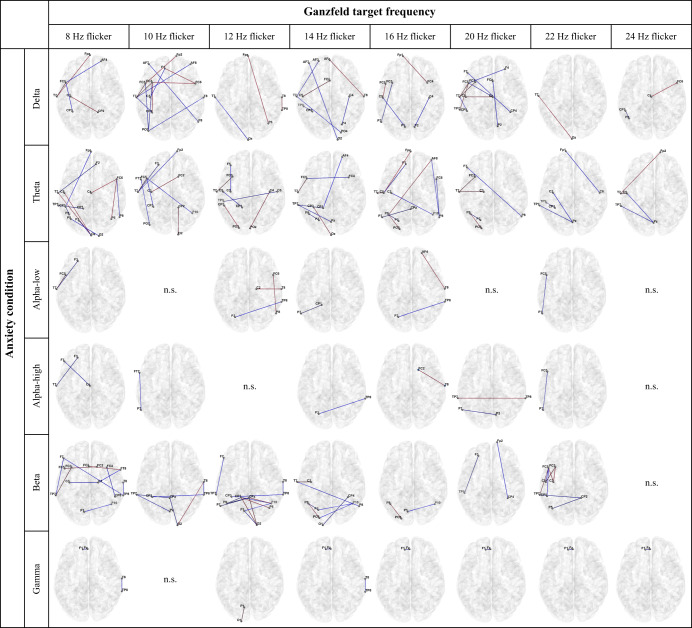
The functional brain networks of significant differences between LS and HS individuals during an anxiety condition for each frequency band (*p* < 0.05, FDR corrected). Note: The Ganzfeld target frequencies are represented in columns, while each row represents one of the frequency bands. The red and blue lines indicate significantly higher and lower functional brain connectivity in HS versus LS individuals, respectively. n.s. indicates no significant difference.

The significant differences in functional brain networks for anxiety conditions between HS and LS groups are shown in Fig. 2 (*p* < 0.05 after FDR correction). The Ganzfeld target frequencies are represented in columns, while each row represents one of the frequency bands. From Fig. 2, a large number of brain connections are significantly different between HS and LS groups during anxiety-induced Ganzfeld condition. Compared with anxiety-induction, the neutral and serenity-induced Ganzfeld conditions show lower significant connections between the two schizotypy groups (see Supplementary Fig. S1).

**Fig. 3:**
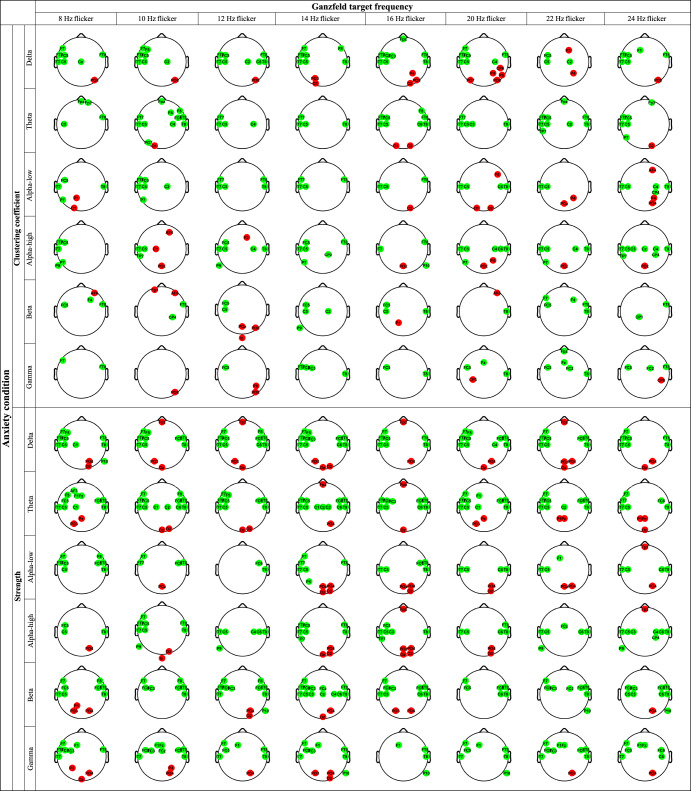
Channels with significant differences between LS and HS individuals during an anxiety condition for each frequency band separately in CC and strength (*p* < 0.05). Note: The Ganzfeld target frequencies are represented in columns, while each row represents one of the frequency bands. The red and green colors indicate that this graph-based index is significantly higher and lower in HS versus LS individuals, respectively.

The channels with significant differences between HS and LS groups during an anxiety condition for graph-based parameters (i.e., CC and strength) are shown in Fig. 3 for each frequency band separately. The target Ganzfeld frequencies are represented in columns, while each row represents one of the frequency bands. As shown in this figure, the LS groups have higher CC and strength than the HS groups. These alterations are observed in bilateral temporal and frontotemporal brain regions. Furthermore, some decreases in CC and strength indices are found in LS groups among occipital and parieto-occipital brain regions. Neutral and serenity-induced Ganzfeld conditions show lower significant connections between the two schizotypy groups compared with anxiety induction (see Supplementary Figs. S2 and S3). Table 2 demonstrates the meaningful statistical differences (*p*-value < 0.001) between HS and LS groups during an anxiety condition for graph-based parameters. Also, the meaningful statistical differences (*p*-value < 0.001) between HS and LS groups during serenity and neutral conditions for graph-based parameters are available in our Supplementary materials (Table S1).

**Table 2:** The meaningful differences (*p*-value < 0.001) of statistical comparison between HS and LS groups during an anxiety condition for CC and strength.

Ganzfeld condition	Frequency	Ganzfeld target frequency (Hz)	Channel	*p*-Value	*t*-Value
**Anxiety (clustering coefficient)**	Delta	8	'FT7'	3.13E−05	−4.679
			'FC5'	0.0002	−4.179
			'T7'	0.0008	−3.654
		10	'FC5'	8.96E−07	−6.266
		12	'FC5'	0.0006	−3.687
			'C5'	4.26E−05	−4.843
			'T7'	0.0001	−4.232
		14	'FT7'	1.66E−07	−6.905
			'FC5'	1.27E−06	−6.137
			'T7'	6.07E−05	− 4.713
		16	'FT7'	2.56E−05	−4.742
			'FC5'	2.22E−06	−5.696
			'C5'	6.68E−06	−5.521
			'T7'	3.24E−05	−4.722
		20	'FT7'	1.12E−04	−4.273
			'T7'	1.51E−05	−5.223
		24	'C5'	5.55E−05	−4.524
			'T7'	4.34E−05	−4.610
	Theta	10	'FT7'	0.0002	−4.043
			'FT8'	0.0005	–3.803
		12	'C5'	5.91E−05	−4.722
			'T7'	0.0004	−3.846
		14	'FT7'	1.97E−04	−4.092
			'C5'	2.41E−04	−4.136
		16	'FT7'	1.32E−05	−4.983
			'C5'	1.47E−05	−5.233
			'T7'	0.0002	−4.119
			'FT8'	1.99E−06	−5.967
		20	'FT7'	0.0003	–3.952
			'C5'	5.87E−05	−4.622
		22	'FT7'	6.48E−04	−3.696
			'C5'	3.64E−04	−3.920
	Alpha_low	10	'FT7'	0.0006	−3.730
		12	'FT8'	7.88E−04	−3.639
		14	'C5'	3.81E−04	−4.036
			'T7'	0.0005	−3.890
		16	'C5'	0.0001	−4.297
			'T7'	0.0005	−3.931
		20	'C5'	0.0004	−3.961
			'T7'	0.0006	−3.810
		22	'T7'	0.0006	–3.841
		24	'T7'	0.0008	–3.716
	Alpha_high	14	'T7'	0.0005	–3.932
		16	'T7'	0.0007	−3.802
		20	'C5'	0.0003	−4.105
		22	'POz'	3.56E−04	4.154
		24	'CP4'	0.0008	−3.642
	Beta	16	'FC5'	0.0001	−4.275
	Gamma	14	'FC3'	0.0007	−3.646
		2	'FC5'	0.0001	−4.397
		24	'FC5'	9.20E−05	−4.561
**Anxiety (strength)**	Delta	8	'F7'	0.0003	−3.991
			'FC5'	0.0001	−4.214
		10	'F7'	0.0001	−4.248
			'FC5'	5.01E−05	−4.565
			'C5'	0.0005	−3.780
			'T8'	0.0006	−3.703
		12	'FT7'	0.0007	−3.647
			'FC5'	4.55E−05	−4.617
			'C5'	2.16E−05	−4.876
			'T7'	5.14E−05	−4.526
			'FT8'	0.0001	−4.165
			'T8'	0.0007	−3.662
		14	'F7'	0.0005	−3.752
			'FT7'	6.42E−05	−4.452
			'FC5'	7.20E−06	−5.172
			'C5'	0.0007	−3.660
			'T7'	0.0003	−3.931
			'Oz'	0.0004	4.035
			'FT8'	0.0001	−4.204
		16	'FC5'	3.15E−05	−4.678
			'C5'	0.0001	−4.320
			'T7'	0.0008	−3.608
			'FT8'	0.0001	−4.182
			'T8'	0.0001	−4.295
		20	'FC5'	1.93E−05	−4.834
			'C5'	4.41E−05	−4.607
			'T7'	0.0001	−4.180
			'FT8'	0.0003	−3.953
		22	'FC5'	0.0006	−3.704
		24	'FC5'	7.33E−05	−4.437
			'C5'	5.50E−05	−4.510
			'T7'	0.0003	−3.948
	Theta	10	'FC6'	9.00E−05	−4.397
		12	'FT7'	0.0006	−3.724
			'FC5'	2.14E−05	−4.799
			'C5'	0.0004	−3.821
		14	'C5'	0.0004	−3.848
			'T7'	0.0005	−3.760
			'FT8'	0.0001	−4.211
			'T8'	0.0006	−3.686
		16	'FC5'	0.0004	−3.845
			'C5'	3.98E−05	−4.617
			'T7'	0.0005	−3.748
			'FT8'	0.0006	−3.739
		20	'FC5'	0.0005	−3.731
			'C5'	0.0005	−3.735
			'FT8'	0.0003	−3.931
	Alpha_low	20	'C5'	6.94E−05	−4.441
	Alpha_high	12	'T8'	0.0006	−3.764
		16	'C5'	0.0007	−3.671
		20	'T7'	0.0008	−3.623
		24	'C5'	0.0001	−4.374
			'T8'	0.0006	−3.736
	Beta	8	'FC5'	0.0004	−3.817
		12	'FC5'	0.0009	−3.624
			'PO4'	0.0009	3.769
		14	'FC5'	0.0002	−3.990
			'C6'	0.0005	−3.748
		16	'FC5'	8.07E−05	−4.425
			'FT8'	0.0001	−4.190
			'T8'	0.0005	−3.776
		22	'FC5'	0.0009	−3.550
	Gamma	22	'FC5'	0.0006	−3.745
		24	'FC5'	0.0007	−3.650

**Fig. 4:**
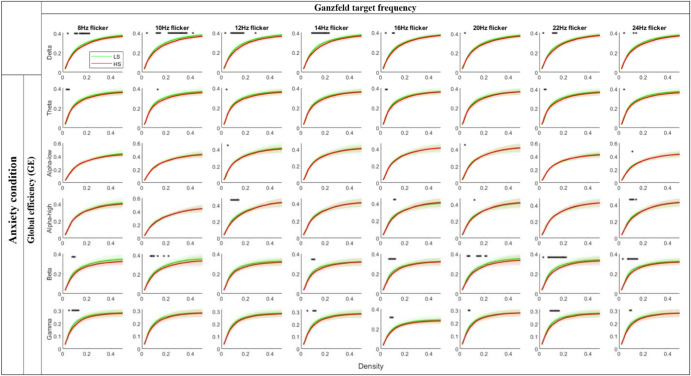
GE of the brain networks as a function of density levels during an anxiety condition for each frequency band for LS (green line) and HS (red line) individuals. Note: The Ganzfeld target frequencies are represented in columns, while each row represents one of the frequency bands. Shaded regions around the mean values indicate the standard deviation; * represents significant differences (*p* < 0.05) between HS and LS individuals.

A comparison of GE in HS and LS groups during an anxiety condition is represented in Fig. 4 as a function of density for each frequency band separately. Each row represents one of the frequency bands, whereas the Ganzfeld target frequencies are shown in columns. Comparing network efficiency in two schizotypy groups shows that GE is significantly different in beta (anxiety, *p-*value = 0.0018, *t*-value = −3.402; serenity, *p-*value = 0.0095, *t*-value = −2.743; neutral, *p-*value = 0.0046, *t*-value = −3.088) and gamma (anxiety, *p-*value = 0.019, *t*-value = −2.453; serenity, *p-*value = 0.007, *t*-value = −2.905; neutral, *p-*value = 0.0166, *t*-value = −2.497) frequency bands in most of these results, while GE is significantly different during anxiety-induced Ganzfeld condition for some density levels in the delta (*p-*value = 0.0049, *t*-value = −3.057) and alpha-high (*p-*value = 0.0117, *t*-value = −2.649) frequency bands (see Supplementary Fig. S4 for serenity and neutral conditions).

### Classification

**Fig. 5:**
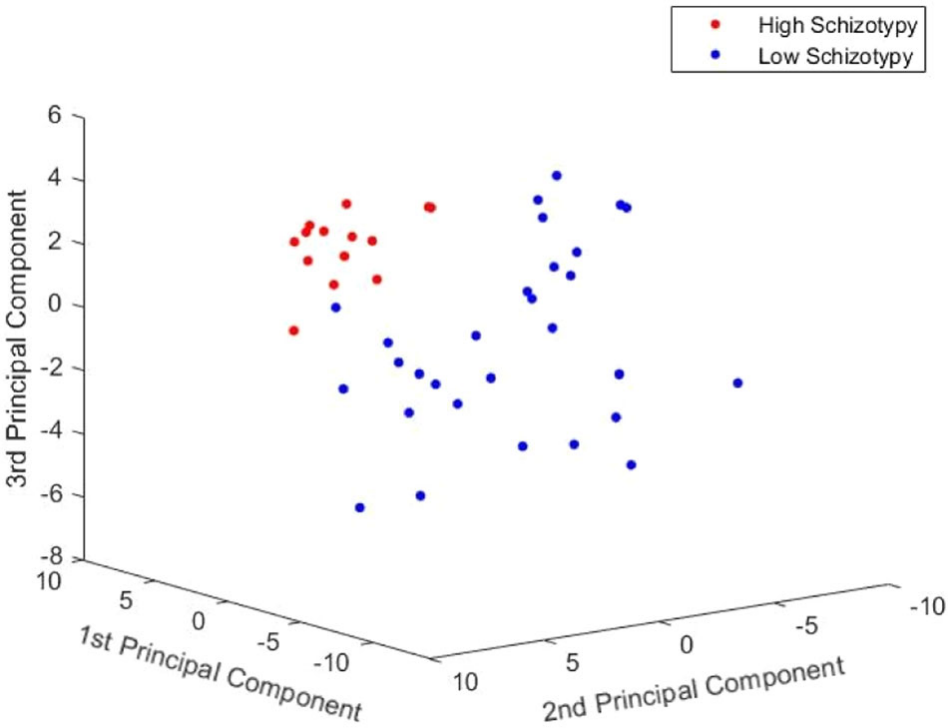
Three-dimensional feature space of the principal components for all participants in the anxiety Ganzfeld condition.

In the present study, the EEG signals from 43 individuals (29 LS and 14 HS) were used to evaluate the machine learning performance using LOO-CV. Collectively, each Ganzfeld condition generates 48 graph-based features (8 Ganzfeld target frequencies times 6 frequency bands) for each EEG channel. It is worth mentioning that the GE features were acquired for the whole brain. After ranking graph-based features using Welch’s *t*-test, a set of features with large *t*-statistic values in the training process was used to evaluate the testing set. These optimal features with lower dimensions are expected to have sufficient discriminative ability to be included in the classification model. The classification performances of the proposed method for each Ganzfeld condition in all graph-based features are shown in Table 3. Among all Ganzfeld conditions, the highest classification performance is achieved by the anxiety-induced Ganzfeld condition vis RUSBoost classifier. In this condition, the accuracy, sensitivity, and specificity are as high as 95.34%, 92.85%, and 96.55%, respectively. Evidently, the serenity Ganzfeld condition with 86.04% accuracy, 86.20% specificity, and 85.71% sensitivity has the second-highest performance in distinguishing between HS and LS individuals using the RUSBoost classifier. Figure 5 presents a three-dimensional feature space of the principal components for all participants in the anxiety Ganzfeld condition. This space was obtained through dimensionality reduction using principal component analysis (PCA). As shown in the figure, the features of the two classes are sufficiently separable.

**Table 3:** The classification performances for different Ganzfeld conditions using different classifiers.

Ganzfeld condition	Classifier	Accuracy	Sensitivity	Specificity	*F*1-score
Neutral	DT	67.44	50	75.86	0.5
	KNN (*K* = 5)	74.41	57.14	82.75	0.59
	LDA	76.74	64.28	82.75	0.64
	Linear-SVM	76.74	64.28	82.75	0.64
	RUSBoost	72.09	78.57	68.96	0.64
Serenity	DT	72.09	50	82.75	0.53
	KNN (*K* = 5)	69.76	71.42	68.96	0.6
	LDA	69.76	57.14	75.86	0.55
	Linear-SVM	74.41	57.14	82.75	0.59
	RUSBoost	86.04	85.71	86.20	0.8
Anxiety	DT	90.69	85.71	93.1	0.85
	KNN (*K* = 5)	90.69	85.71	93.1	0.85
	LDA	93.02	85.71	96.55	0.88
	Linear-SVM	93.02	85.71	96.55	0.88
	RUSBoost	**95.34**	**92.85**	**96.55**	**0.92**

The best performances are highlighted in bold.

## Discussion

For the first time (to the best of our knowledge), the current study examines the relationship between weighted functional brain networks under Ganzfeld conditions as a function of positive schizotypy. Graph theory parameters derived from the PLV adjacency matrix were used to accurately classify brain states using a machine learning approach.

The two schizotypy groups were accurately differentiated based on functional brain networks, especially in delta, theta, and beta frequency bands and during anxiety mood-induction conditions. Compared to the LS individuals, phase synchrony was decreased in the HS individuals in most connections, although some increases in functional brain networks of HS individuals were observed. Such results are in line with those studies in the first episode of psychosis, which show diffuse connectivity patterns and lack of synchronization between brain regions^22,27,51^. Furthermore, abnormal functional connectivity in the anterior cingulate cortices has previously been found during hallucinations^52,53^. Similar to schizophrenia symptoms, these deficits possibly indicate a decrease in regional computation processing or insufficient information flow between specific cortical regions^28^. In other words, decreases in brain connectivity might represent an absence of synchrony between brain regions^22^.

LS groups generally have higher CC and strength than HS groups. However, these differences were most prominent in bilateral temporal and frontotemporal, as well as medial frontal regions. This is in line with theories indicating the importance of frontal and temporal regions in psychosis^54,55^ that extend to schizotypy^17,54,56,57^. Furthermore, the finding that anxiety and neutral-induced Ganzfeld stimulation were shown to have the most and the slightest differences between groups, respectively, points to the role of emotion in schizotypy.

In comparison, other regions showed greater CC and strength indices in HS groups among prefrontal (anxiety and serenity conditions only), occipital, and parieto-occipital brain regions. Prefrontal connectivity might reflect a hypersensitivity to affect in the HS group. Future studies might investigate whether the opposite occurs in relation to negative schizotypy, which is often characterized by emotional blunting and anhedonia. The effect at posterior sites is in line with the proposal of a hyperactive occipital cortex under Ganzfeld conditions in relation to schizotypy^4^.

Despite some support for the disconnection hypothesis in schizophrenia, there have been inconsistent findings in the studies of schizotypy^16,22,23,27^. Similar to schizophrenia, some schizotypy studies demonstrate reduced brain connectivity^16,22,27^. In comparison, greater structural connectivity, measured using diffusion tensor MRI, in HS individuals was previously interpreted as reflecting a protective mechanism against developing pathology^23^. Alternatively, degeneration of myelinated axons might reflect illness-related damage rather than cause^23^. Furthermore, Messaritaki and colleagues observed that the HS individuals showed higher degrees and CC in the default mode network (DMN) and sensorimotor areas^23^. This divergence of results between our study and Messaritaki et al.^23^. may be due to not only the differences between the resting state used by Messaritaki and colleagues and the current Ganzfeld conditions but also the differences in the selected areas in DMN and sensorimotor. It should be mentioned that our results in some brain regions, including frontal, occipital, and parieto-occipital are in line with the study by Messaritaki et al. for CC and strength indices. It may also be due to the correlation between these brain regions and the related areas in DMN.

Finally, our machine learning results show that anxiety-induced Ganzfeld stimulation is a reliable task for accurate diagnosis of HS individuals in the general population. Figure 5 presents compelling evidence supporting the assertion that features extracted from graph-based measures are highly distinct. These distinctive features can be leveraged to design a reliable diagnostic system during anxiety-induced Ganzfeld stimulation. To achieve optimal classification rates, several well-known classifiers were applied to the extracted features. As can be seen from Table 2, the RUSBoost classifier has the best performance among all the classifiers. These findings reveal that our ensemble classifier correctly diagnoses the severity of schizotypy in individuals. This may prove the claim that this algorithm is a powerful classification method for imbalanced data.

Some crucial limitations of the present study should be declared. Our analysis was based on forty-three positive schizotypy individuals. So, future studies should consider not only a larger number of participants but also balanced data in sex, age, and data samples. Future studies may benefit from involving other important demographics, such as ethnicity and education level. Considering the cross-cultural differences in brain activities, it would be beneficial to consider more individuals with different ethnic backgrounds to improve the system’s reliability and robustness to be suitable as a clinical diagnostic tool. In addition to ethnicity, exploring the impact of other demographic risk factors, including a history of psychological trauma, cannabis use, urbanicity, and social isolation, could be promising directions for future research. Selecting an optimal threshold is one of the most critical steps in graph theory studies. To enhance the reliability and robustness of the results, we suggest that, for future studies, a more suitable thresholding technique is developed. EEG network analysis can be influenced by the reference choice^58,59^. To improve the validity and robustness of future studies, it is recommended to use advanced re-referencing techniques such as the reference electrode standardization technique (REST)^60^. Finally, other measures of brain complexity, such as entropy, should be considered in future studies.

In conclusion, the present study is the first of its kind to investigate the weighted functional brain network in positive schizotypy individuals during Ganzfeld-induced hallucinatory imagery stimulation. We examined some graph network parameters for an accurate diagnosis of HS and LS individuals using a machine learning method. These findings show that anxiety-induced Ganzfeld stimulation has the most vital and discriminatory role among three Ganzfeld conditions in schizotypy classification based on graph parameters. The highest difference between HS and LS groups was observed in bilateral temporal and frontotemporal, occipital, and parieto-occipital brain regions. HS groups showed lower CC and strength values in both temporal and frontotemporal brain regions while revealing higher values of these measures in the occipital and parieto-occipital brain regions than LS groups. Considering integration measures, HS groups in almost all frequency bands showed lower GE than LS groups. These outcomes are very useful in assessing schizotypy in its early stage of development. Therefore, our study may provide new insights into an accurate diagnosis of the first episode of psychosis based on brain networks during the pseudo-hallucinatory imagery task.

### Supplementary information

Supplementary material

## Data Availability

The data are not publicly available for ethical reasons but could be available from the corresponding author upon reasonable request.

## Code availability

The data were preprocessed using the EEGLAB toolbox (https://sccn.ucsd.edu/eeglab/index.php) running on MATLAB 2019a (The Mathworks, Inc). The Brain Connectivity Toolbox (www.brain-connectivity-toolbox.net) was used to estimate graph measures. Also, the visualization of brain space graphs was performed using the BrainNet Viewer toolbox (https://www.nitrc.org/projects/bnv/). The code for data processing in this study is available from the corresponding author upon reasonable request.
