# Supplementary material for: Graph-based analysis of EEG for schizotypy classification applying flicker Ganzfeld stimulation
Source: Schizophrenia (Heidelb). 2023 Sep 21;9(1):64. doi: 10.1038/s41537-023-00395-4 (PMC10514040; doi:10.1038/s41537-023-00395-4)
Supplement: Supplementary file 1 — Supplementary material [file 41537_2023_395_MOESM1_ESM.docx]

**Supplementary materials**

Title: **Graph-based Analysis of EEG for Schizotypy Classification Applying Flicker Ganzfeld Stimulation**

**Supplementary tables and figures**


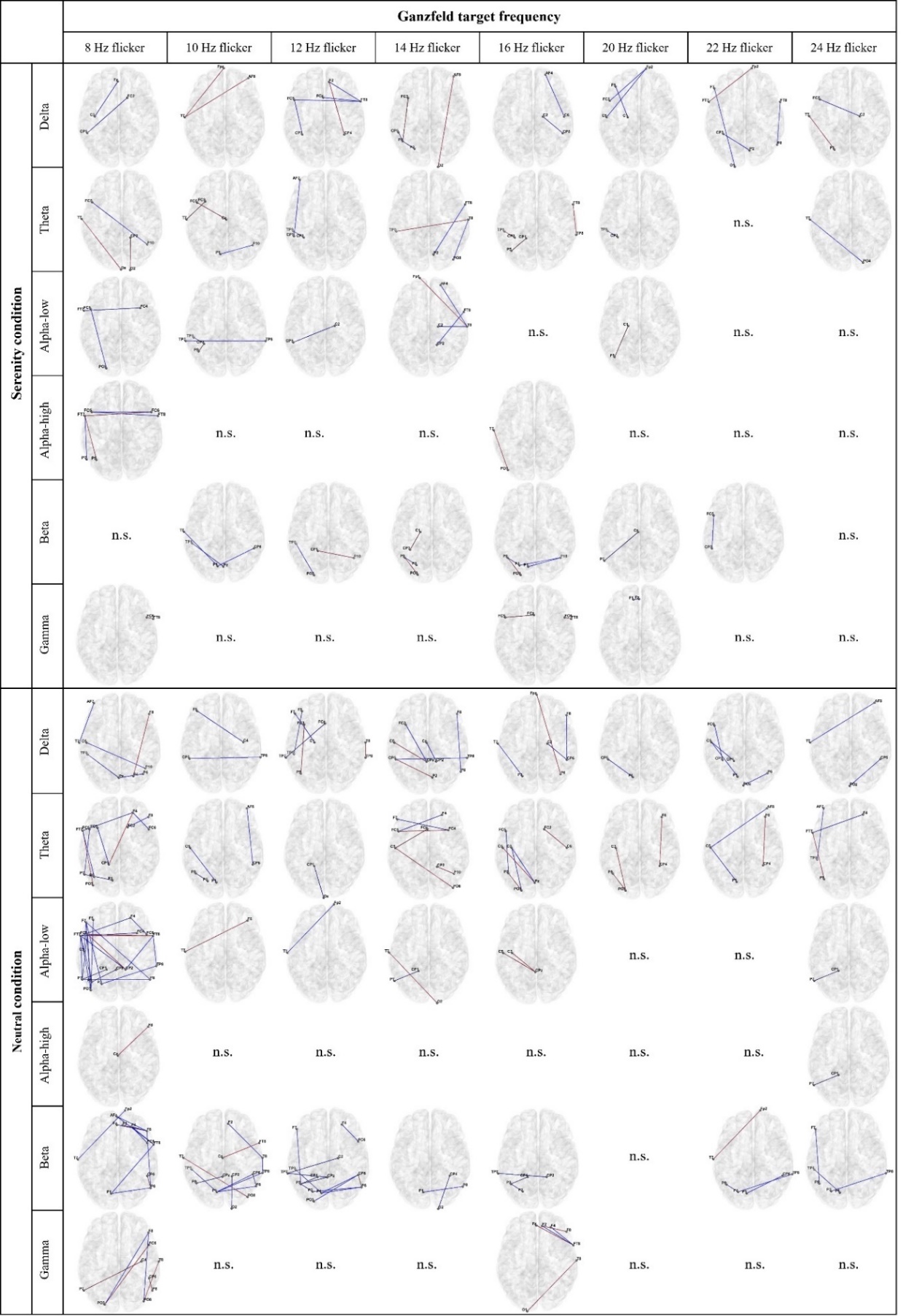


Figure S1. The functional brain networks of significant differences between LS and HS individuals in six frequency bands (i.e., delta, theta, alpha-low, alpha-high, beta, and gamma) for serenity and neutral conditions (*p* < 0.05, FDR corrected). Note: The Ganzfeld target frequencies are represented in columns, while each row represents one of the frequency bands. The red and blue lines indicate significantly higher and lower functional brain connectivity in HS versus LS individuals, respectively. n.s. indicates no significant difference.


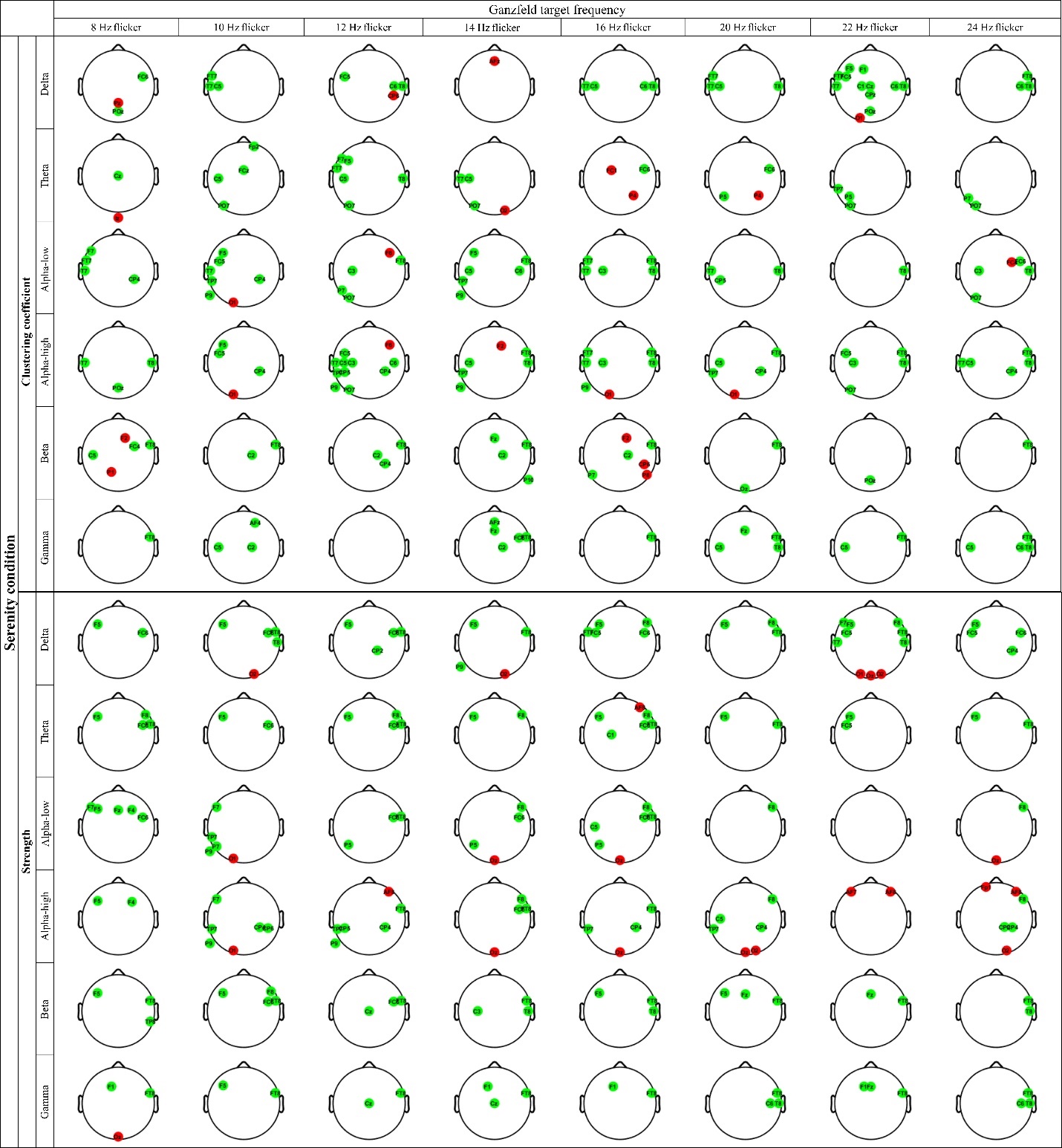


Figure S2. Channels with significant differences in CC and strength between LS and HS individuals during a serenity condition for each frequency band separately (*p* < 0.05). Note: The Ganzfeld target frequencies are represented in columns, while each row represents one of the frequency bands. The red and blue colors indicate that this graph-based index is significantly higher and lower in HS versus LS individuals, respectively.


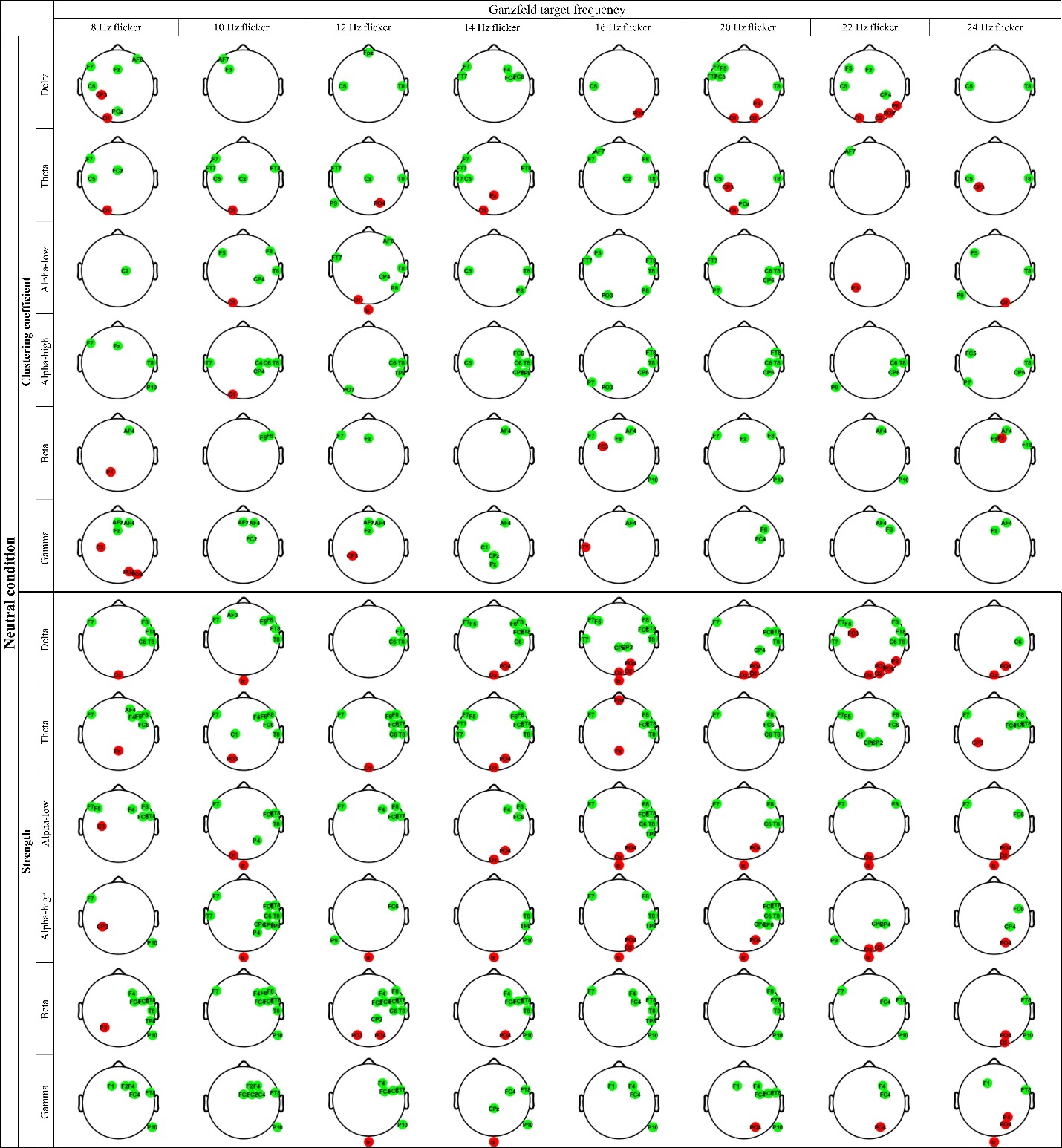


Figure S3. Channels with significant differences in CC and strength between LS and HS individuals during a neutral condition for each frequency band separately (*p* < 0.05). Note: The Ganzfeld target frequencies are represented in columns, while each row represents one of the frequency bands. The red and blue colors indicate that this graph-based index is significantly higher and lower in HS versus LS individuals, respectively.


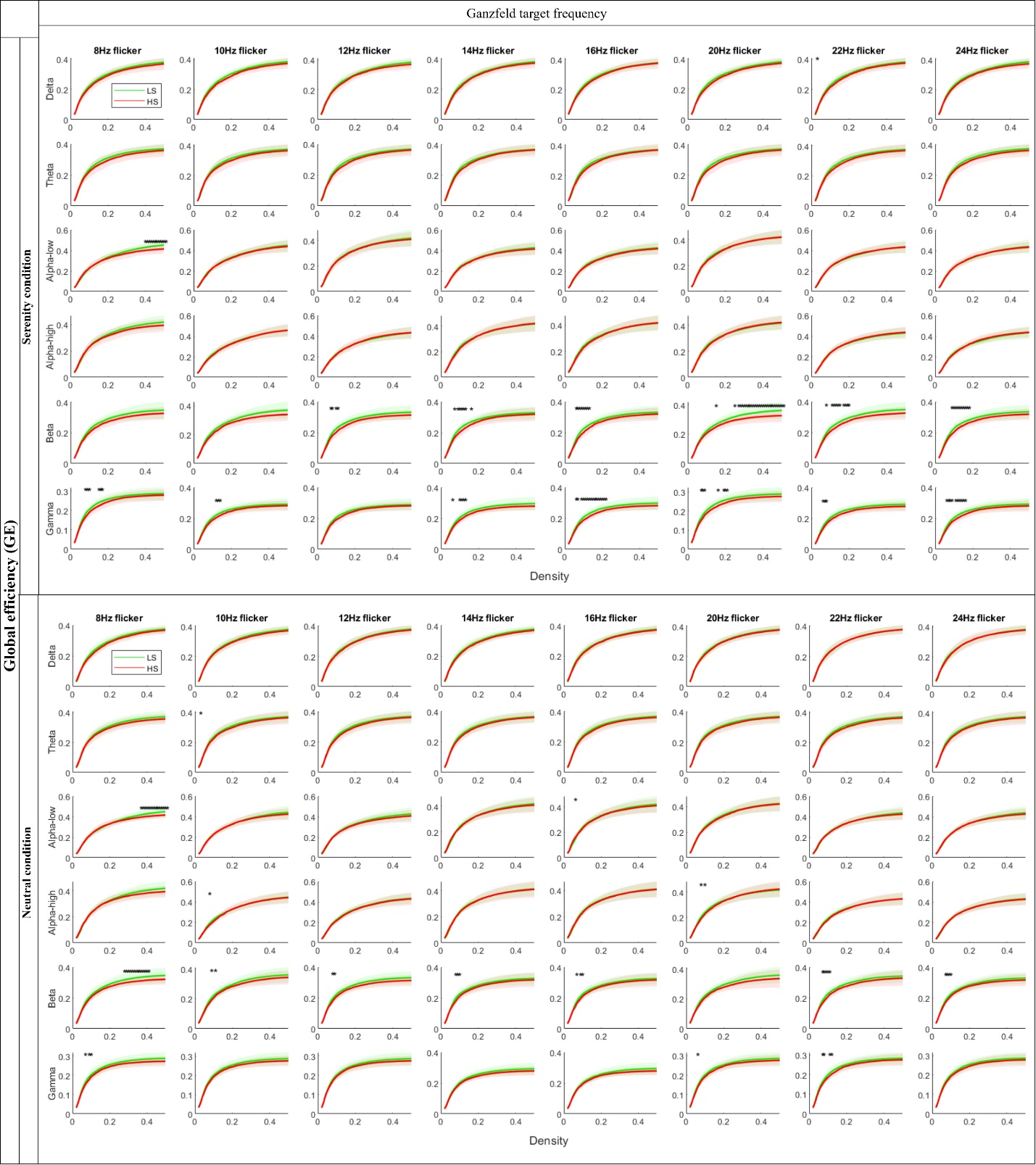


Figure S4. GE of the brain networks as a function of density levels in six frequency bands for LS (green line) and HS (red line) individuals during the serenity and neutral conditions. Notes: Shaded regions around the mean values indicate the standard deviation; * represents significant differences (*p* < 0.05) between HS and LS individuals.

Table S1. the meaningful differences (p-value < 0.001) of statistical comparison between HS and LS groups during serenity and neutral conditions for CC and strength.

| Ganzfeld condition | Frequency | Ganzfeld  target frequency | Channel | *p*-value | *t*-value |
| --- | --- | --- | --- | --- | --- |
| Serenity (clustering coefficient) | Delta | 20 HZ | 'T7' | 0.0002 | -3.999 |
|  | Alpha_low | 16 HZ | 'FT8' | 0.0003 | -3.934 |
|  | Alpha_high | 14 HZ | 'FT8' | 0.0003 | -3.953 |
|  |  | 24 HZ | 'CP4' | 0.0008 | -3.754 |
|  | Gamma | 14 HZ | 'C2' | 0.0008 | -3.669 |
|  |  | 24 HZ | 'C5' | 0.0006 | -3.697 |
| Serenity (strength) | Delta | 16 HZ | 'F5' | 0.0008 | -3.639 |
|  | Beta | 14 HZ | 'FT8' | 0.0002 | -4.076 |
|  |  | 16 HZ | 'FT8' | 0.0001 | -4.286 |
|  |  | 24 HZ | 'FT8' | 0.0004 | -3.788 |
|  | Gamma | 8 HZ | 'FT8' | 0.0009 | -3.546 |
|  |  | 14 HZ | 'FT8' | 0.0003 | -3.940 |
|  |  | 16 HZ | 'FT8' | 0.0005 | -3.734 |
|  |  | 24 HZ | 'FT8' | 0.0002 | -4.075 |
| Neutral (clustering coefficient) | Theta | 20 HZ | 'T8' | 0.0004 | -3.950 |
|  | Alpha_high | 20 HZ | 'CP6' | 0.0003 | -3.968 |
| Neutral (strength) | Delta | 16 HZ | 'F7' | 0.0008 | -3.615 |
|  |  |  | 'FT8' | 4.55E-05 | -4.570 |
|  |  | 20 HZ | 'FT8' | 0.0007 | -3.650 |
|  | Theta | 12 HZ | 'F7' | 0.0005 | -3.799 |
|  |  |  | 'FC6' | 0.0007 | -3.655 |
|  |  | 14 HZ | 'FC6' | 0.0008 | -3.599 |
|  | Beta | 12 HZ | 'FC4' | 0.0005 | -3.893 |
